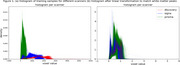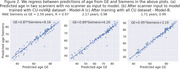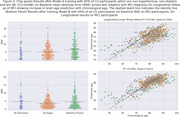# Developing scanner change invariant brain age models for aging and dementia studies

**DOI:** 10.1002/alz70856_097891

**Published:** 2025-12-24

**Authors:** Siddhartha Satpathi, Robel K Gebre, Jeffrey L. Gunter, Scott A. Przybelski, Matthew L. Senjem, Kejal Kantarci, Clifford R. Jack, Prashanthi Vemuri

**Affiliations:** ^1^ Department of Radiology, Mayo Clinic, Rochester, MN, USA; ^2^ Department of Quantitative Health Sciences, Mayo Clinic, Rochester, MN, USA; ^3^ Department of Information Technology, Mayo Clinic, Rochester, MN, USA

## Abstract

**Background:**

Brain age models are increasingly being used as measures of brain health and neurodegeneration. These models can be deep learning (DL) based and/or classical methods and provide a brain age gap (BAG) which is the difference between chronological and predicted brain age. Our goal was to develop DL‐based scanner invariant brain age models that can be applied in aging and dementia studies even when there are scanner changes that may hinder longitudinal follow‐up.

**Method:**

We identified 3374 cognitively unimpaired (CU) participants with T1 scans from the Mayo Clinic Study of Aging (MCSA). A subset of 863 CU amyloidosis negative (PiB‐PET cutoff < 25 Centiloid) and no history of diabetes and hypertension was selected (CU‐noVAβ). We built two models – Model‐A trained on CU‐noVAβ, and Model‐B trained on all CU. We included 453 MCSA MCI participants as test set for both models. We used 113 participants (74 CU, 28 MCI, 10 dementia) from MCSA and Mayo ADRC with scans from two different vendors (scanned within a week of each other) for testing cross‐vendor/scanner differences (cross‐vendor data). The two unique features of our model included histogram matching and scanner type as an input to the DenseNet model for training (Figure 1).

**Result:**

In the data, the inclusion of scanner as an input decreased the prediction of mean age differences between the scanners (Model‐A=2.17 years; Model‐B=1.71 years) (Figure 2). The mean absolute error (MAE=absolute value of BAG) for Model‐A in MCI was 5.72 years and Model‐B in MCI was 4.06 years, consistent across scanners (Figure 3). Longitudinal prediction increased with age (Model‐A slope=0.53; Model‐B slope=0.63).

**Conclusion:**

We found that training on all CU (Model B) rather than a subset CU‐noVAβ (Model A) allowed for the DenseNet model to backpropagate on more variable data which lowered MAE in prediction error between scanners and lowered MAE for MCIs. Histogram matching and scanner as an input to the brain age model accounted for scanner changes and resulted in scanner invariant longitudinal brain age estimation.